# Complete genome sequences of agricultural azole-resistant *Penicillium rubens* encoding CYP51A and ERG11 paralogues

**DOI:** 10.1128/MRA.00188-23

**Published:** 2023-09-01

**Authors:** Jeff Gauthier, Sima Mohammadi, Jesse Huffman, Opeyemi U. Lawal, Irena Kukavica-Ibrulj, Marianne Potvin, Lawrence Goodridge, Roger C. Levesque

**Affiliations:** 1 Institut de biologie intégrative et des systèmes (IBIS), Faculté de médecine, Université Laval, Québec, Canada; 2 Canadian Research Institute for Food Safety, Department of Food Science, University of Guelph, Ontario, Canada; University of California, Riverside, Riverside, California, USA

**Keywords:** azole resistance, antifungal resistance, *Penicillium*, agriculture, antifungal agents, food-borne pathogens

## Abstract

Azoles are major antifungals in agriculture and medicine. However, the surge of intrinsic azole resistance is critical for public health. Here, we present the complete long-read sequencing of three azole-resistant *Penicillium rubens* from food crops. The presence of CYP51A and ERG11 paralogues was confirmed, as in other azole-resistant *P. rubens*.

## ANNOUNCEMENT

Azoles have been used extensively since the mid-1960s to control the growth of fungi in agriculture (1). However, their use fueled the surge of antifungal resistance (2), which is a top-priority threat for global human health, according to the World Health Organization (3). One azole resistance mechanism in fungi is the acquisition of lanosterol demethylase (CYP51A) (4) and lanosterol 14-alpha-demethylase (ERG11) paralogues (5). Therefore, we sequenced and assembled the genome of three agricultural azole-resistant *Penicillium rubens* isolates with Oxford Nanopore long-read DNA technology: MT45, from Mexican tomatoes; WT45, from Ontario (Canada) wheat; and BM32, from Ontario (Canada) barley.

Vegetable samples were homogenized, and serial dilutions of the homogenate were individually plated on Potato Dextrose Agar plates supplemented with difenoconazole (30 μg/mL) and incubated for 5 days at 25°C. DNA was extracted with the PowerSoil DNA Kit (QIAGEN) and quantified using the Qubit dsDNA BR method (Thermo Fisher). DNA libraries were then prepared with the Ligation Sequencing Kit (Oxford Nanopore, SQK- LSK109) and then loaded on R9.4.1 flowcells in a GridION apparatus. Reads were basecalled and demultiplexed using Oxford Nanopore’s Guppy basecaller v6.4.6 (see Table 1 for read statistics). In addition, reads were filtered and trimmed with NanoFilt v2.3.0 (https://github.com/wdecoster/nanofilt) to select reads with a Phred quality score of 10 and above plus a minimum length of 1,000 bp. Assemblies were then made with CANU v2.1 (https://github.com/marbl/canu) and corrected with Medaka v1.4.3 (https://github.com/nanoporetech/medaka). For each assembly, taxonomy was assigned with Kraken v2.1.2 (https://github.com/DerrickWood/kraken2) against a local build of the standard Kraken2 database (compiled on 25 January 2023). Genome completeness was assessed with BUSCO v5.2.2 (https://gitlab.com/ezlab/busco) and the ODB10 Ascomycota data set. All software used were run with default parameters.

**TABLE 1:** Main assembly and coverage metrics for *Penicillium rubens* MT45, WT45, and BM32

Strain	*P. rubens* MT45	*P. rubens* WT45	*P. rubens* BM32	*P. rubens* Wisconsin 54-1255 (reference)*^a^*
Assemblies				
Estimated genome size (bp)	28,647,743	28,746,596	28,713,308	32,223,735
Longest (bp)	7,524,203	7,524,624	7,524,050	6,387,817
Contig N50 (bp)	4,505,778	4,506,134	4,505,581	3,889,175
Contigs ≥N50	3	3	3	4
Contig N90 (bp)	3,002,355	3,002,608	3,002,242	709,213
Contigs ≥N90	6	6	6	8
Shortest (bp)	55,978	42,108	5,484	1,032
Number of contigs	7	8	10	49
GC content (%)	46.4%	47.3%	45.1%	48.1%
*N* count^*b*^	0	0	0	19,918
Gaps	0	0	0	775
Nanopore reads				
Number of reads	209,419	648,293	250,434	Unavailable
Total read bases (bp)	1,418,937,418	5,550,803,731	1,332,762,391	Unavailable
Read N50 (bp)	10,419	12,917	10,499	Unavailable
Read N90 (bp)	3,986	4,861	2,864	Unavailable
Mean coverage (×)	50	193	46	Unavailable
Completeness				
Completeness score (%)	96.5	97.0	96.3	97.4
Complete one-copy BUSCOs	1,632	1,651	1,631	1,648
Complete duplicated BUSCOs	13	4	12	13
Fragmented BUSCOs	11	8	10	6
Missing BUSCOs	50	43	53	39
Total BUSCO groups searched	1,706	1,706	1,706	1,706

^*a*^ Assembly metrics were calculated from data retrieved on the NCBI Genome and SRA databases. This genome (BioProject PRJNA39879) is also the only entry for *P. rubens* on RefSeq. Raw reads were not publicly available for this BioProject.

^*b*^ *N* count, number of ambiguous nucleotides in the genome.

All assemblies had an estimated genome size of 28.70 ± 0.04 Mbp with an N50 size of 4.506 ± 0.002 Mbp. *Penicillium rubens* MT45, WT45, and BM32, respectively, had 7, 8, and 10 contigs (Table 1). For all three genomes, taxonomy was assigned by Kraken2 to *Penicillium rubens*, with *P. rubens* Wisconsin 54-1255 as the “last common ancestor” strain. Furthermore, completeness ranged from 96.3% to 97.0% for all three genomes, as for reference genome *P. rubens* Wisconsin 54-1255 (Table 1). In comparison with 54-1255, the assemblies of MT45, WT45, and BM32 were devoid of unknown bases and gaps while also being more contiguous (7–10 contigs versus 49). Furthermore, there were six contigs above or equal to the N90 size (~3.0 Mbp), suggesting that their assembly is chromosome level.

Interestingly, the presence of CYP51A and ERG11 homologs was verified by BLASTX search (version 2.13.0). Matching gene sequences were then compared to putative orthologs in MT45, WT45, and BM32 by multiple sequence-translated nucleotide alignment with MUSCLE v3.4.31 (https://drive5.com/muscle) and TranslatorX (http://translatorx.co.uk). In both cases, a large portion of the N-terminal domain was missing in comparison to wild-type sequences (AGH55425.1 for CYP51A and KAA8642166 for ERG11, respectively). Whether these paralogues confer resistance to azoles is currently under investigation.

## Data Availability

This Whole Genome Shotgun sequencing project has been deposited under the following number: PRJNA932827. The versions of BM32, MT45, and WT45 described in this paper are the first versions, respectively, GCA_029142745.1, GCA_029142755.1, and GCA_029142805.1. Raw reads were submitted to the NCBI SRA under the following accessions for BM32, MT45, and WT45, respectively: SRR23456031, SRR23456033, and SRR23456032.
